# Expression and 7-day time course of circulating microRNAs in septic patients treated with nephrotoxic antibiotic agents

**DOI:** 10.1186/s12882-022-02726-6

**Published:** 2022-03-19

**Authors:** Nadezda Petejova, Arnost Martinek, Josef Zadrazil, Viktor Klementa, Lenka Pribylova, Radim Bris, Marcela Kanova, Radka Sigutova, Ivana Kacirova, Zdenek Svagera, Eva Bace, David Stejskal

**Affiliations:** 1 Department of Internal Medicine and Cardiology, University Hospital Ostrava, 70852 Ostrava, Czech Republic; 2 Faculty of Medicine, University of Ostrava, 70300 Ostrava, Czech Republic; 3 Department of Internal Medicine III—Nephrology, Rheumatology and Endocrinology, Faculty of Medicine and Dentistry, Palacky University Olomouc, 77900 Olomouc, Czech Republic; 4 Department of Internal Medicine III—Nephrology, Rheumatology and Endocrinology, University Hospital Olomouc, 77900 Olomouc, Czech Republic; 5 Department of Applied Mathematics, Faculty of Electrical Engineering and Computer Science, VSB Technical University of Ostrava, 70800 Ostrava, Czech Republic; 6 Department of Anesthesiology and Resuscitation, University Hospital Ostrava, 70852 Ostrava, Czech Republic; 7 Department of Epidemiology and Public Health, Faculty of Medicine, University of Ostrava, 70300 Ostrava, Czech Republic; 8 Department of Laboratory Diagnostics Institute of Clinical Biochemistry and Clinical Pharmacology, University Hospital Ostrava, 70852 Ostrava, Czech Republic; 9 Department of Immunodiagnostics, BioVendor R&D, Laboratory Medicine Corp, 621 00 Brno, Czech Republic

**Keywords:** Acute kidney injury, Gentamicin, microRNA, Nephrotoxicity, Sepsis, Vancomycin

## Abstract

**Background:**

Through regulation of signaling pathways, microRNAs (miRNAs) can be involved in sepsis and associated organ dysfunction. The aims of this study were to track the 7-day time course of blood miRNAs in patients with sepsis treated with vancomycin, gentamicin, or a non-nephrotoxic antibiotic and miRNA associations with neutrophil gelatinase-associated lipokalin (NGAL), creatinine, procalcitonin, interleukin-6, and acute kidney injury (AKI) stage.

**Methods:**

Of 46 adult patients, 7 were on vancomycin, 20 on gentamicin, and 19 on another antibiotic. Blood samples were collected on days 1, 4, and 7 of treatment, and miRNAs were identified using quantitative reverse transcription PCR.

**Results:**

The results showed no relationship between miRNA levels and biochemical variables on day 1. By day 7 of gentamicin treatment *miR-15a-5p* provided good discrimination between AKI and non-AKI (area under curve, 0.828). In patients taking vancomycin, *miR-155-5p* and *miR-192-5p* positively correlated with creatinine and NGAL values, and *miR-192-5p* and *miR-423-5p* positively correlated with procalcitonin and interleukin-6 in patients treated with a non-nephrotoxic antibiotic. In patients together we found positive correlation between *miR-155-5p* and *miR-423-5p* and all biochemical markers.

**Conclusion:**

The results suggest that these four miRNAs may serve as diagnostic or therapeutic tool in sepsis, renal injury and nephrotoxic treatment.

**Trial registration:**

ClinicalTrials.gov, ID: NCT04991376. Registered on 27 July 2021.

## Background

Sepsis is generally defined as a life-threatening and dysregulated reaction to infection leading to systemic inflammation and multiple organ dysfunction in the worst-case scenario [1].

More than 40% of critically ill hospitalized patients with acute kidney injury (AKI) in intensive care units (ICUs) have sepsis or septic shock [2]. The overall incidence of AKI is 10 to 15% in hospitalized patients and more than 50% in ICUs [3]. AKI in critically ill patients is a common and usually serious condition associated with increased patient morbidity and mortality. Depending on the stage of renal damage, AKI is often linked to decreased diuresis, leading to a volume overload and exacerbations in critically ill patients [4]. AKI is classified globally based on two parameters, increased serum creatinine (S_crea_) and decreased urine output, as established by the Kidney Disease Improving Global Outcome (KDIGO) association in 2012 [5]. The pathophysiology of sepsis-induced AKI is considered to be multifactorial, with damage to almost all parts of the renal parenchyma at various levels, from the renal macro- and microcirculation to cycle cell arrest and lethal injury of renal epithelial cells [6]. From a clinical perspective, worsening of kidney function to KDIGO stage 2 or 3 usually indicates the need to initiate renal replacement therapy (RRT) and carries increased risk for in-hospital mortality and chronic dialysis [7]. In addition, effective antimicrobial treatment is crucial in sepsis therapy, but the risk of renal injury increases with the use of these lifesaving but often potentially nephrotoxic treatments, as does development of ischemia via organ hypoperfusion and inflammation in sepsis. Drug-induced nephrotoxicity from these antimicrobial treatments varies from a relatively mild form of acute interstitial nephritis (with, e.g., penicillins, cephalosporins, macrolides, antiretrovirals, and fluoroquinolones) to severe renal damage, as indicated by acute tubular necrosis and a frequent need for RRT (with, e.g., vancomycin, aminoglycosides, or polymyxins) [8]. To prevent nephrotoxicity and avoid subtherapeutic dosing, drug serum concentrations are usually monitored, with adjustment of dose or dosing interval according to pharmacokinetic models.

Vancomycin is a glycopeptidic antibiotic with effects that depend on time and area under the curve (AUC) above the minimum inhibitory concentration (MIC) of the pathogen. It exerts bactericidal effects on Gram-positive bacteria, including methicillin-resistant *Staphylococcus aureus*. The most useful parameter for detecting the effects of vancomycin treatment is a 24-h AUC:MIC ratio ≥ 400 mg x h/L with a pathogen MIC< 2 mg/L [9, 10]. Vancomycin nephrotoxicity not only tracks with total administered dose but also can be worsened by other concomitant therapy, patient age, and trough serum vancomycin > 16.5 mg/L [11]. Gentamicin is a concentration-dependent aminoglycoside antibiotic that is effective against Gram-negative bacteria such as *Escherichia coli*, *Pseudomonas aeruginosa*, and *Klebsiella* sp. The pathophysiological mechanism of its nephrotoxicity involves many factors, including direct tubular, glomerular, or vascular damage with activation of oxidative stress. Gentamicin is eliminated by glomerular filtration and partially undergoes tubular resorption with accumulation in cytoplasm and lysosomes with activation of cell death [12].

In the quest for new preventive or therapeutic targets in septic/nephrotoxic acute renal damage, a current research focus is on the small RNAs known as micro-RNAs (miRNAs or miRs, 18–31 nucleotides). Their clinical significance varies because they can positively or negatively regulate a substantial number of target genes. Many miRNAs have been investigated in animal and human studies, including in blood, urine, or sweat, for potential use as biomarkers of sepsis. Among critically ill patients, sepsis and the non-septic systemic inflammatory response (SIRS) are associated with substantial and differential changes in circulating miRNAs [13]. In sepsis, miRNAs can critically affect the innate and acquired immunity systems. Many of these molecules influence biochemical pathways associated with NF-kB activation and production of pro-inflammatory cytokines such as tumor necrosis factor alpha (TNF-α), interleukin (IL)-6, IL-1β, IL-12, and IL-10 [14, 15].

Studies have confirmed the impact of miRNAs involved in the nephrotoxic effects of vancomycin on renal cell apoptosis via p53 regulation or DNA methylation on HK-2 cells and mice renal cortical tissue [16, 17]. Gentamicin nephrotoxicity has been associated with rats urine miRNAs linked to regulation of genes and their apoptosis and cycle cell arrest signaling pathways, including *mitogen-activated protein (MAP) kinase* (*MAPK*), *P13K/AKT*, *Ras*, *p53*, and *FoxO* [18]. The other miRNAs investigated in urine in rats are reported to be involved in the *Ras*, *MAP2K1*, myeloid leukemia cell differentiation protein 1, transgelin, and vimentin signaling pathways with activation of renal tubular cell necrosis in the worst stage [19].

Based on data from previously published experimental, animal and human studies, we identified 20 miRNAs associated with biochemical signaling pathways involved in inflammation, organ ischemia, and drug (vancomycin/gentamicin) nephrotoxicity - review [14, 15], Chen et al. 2016 (mice renal cortical tissue and HK-2 cells) [16], Wang et al. 2018 (mice renal cortical tissue and HK-2 cells) [17], Zhou et al. 2016 (rats urine) [18], Nassirpour et al. 2014 (rats urine) [19], Ge at al 2017 (human blood/serum) [20], Schlosser et al. 2015 (human blood/plasma) [21], Saikumar et al. 2012 (rats urine, blood and renal tissue and human urine sample and renal biopsy) [22], Tacke et al. 2014 (human blood/serum) [23]. These differentially expressed circulating miRNAs have been linked to septic, ischemic, or nephrotoxic AKI in critically ill patients with sepsis. In this study, our first aim was to determine and investigate specific blood miRNAs with the most significant expression in terms of specificity and yield of selected miRNA sequences in patients with sepsis, tracking changes in miRNA expression over 7 days of treatment with nephrotoxic (vancomycin or gentamicin) as compared with other, non-nephrotoxic antibiotic treatment (oATB). Our second aim was to determine at day 7 the association between circulating miRNA expression and changes in serum neutrophil gelatinase–associated lipocalin (NGAL) [24], S_crea_ [5], the inflammatory markers procalcitonin (PCT) and IL-6 [25], and AKI stage [5] to assess the potential of these small RNAs as pathophysiological markers or therapeutic targets in septic patients with or without AKI.

## Materials & methods

### Study design, subjects & ethics information

For this study, we adhered to the Declaration of Helsinki, 2013, Good Clinical Practice, and obtained approval from the institutional Ethics Review Board of both University Hospital Ostrava (Reference number 55/2019, protocol code number *III*) and University Hospital Olomouc (Reference number 13/19), Czech Republic. Written informed consent was obtained from all patients involved in the study.

This prospective open clinical study included an initial 53 critically ill adult patients with sepsis, hospitalized in a general ICU or in an intermediate care at the university hospitals of Ostrava and Olomouc in 2019 and 2020. All patients suffered from sepsis (defined below) and were treated with vancomycin or gentamicin in combination with other not-severely nephrotoxic antibiotics or with oATBs alone. For the statistical analysis, we used data from 46 patients with completed miRNA and NGAL values: 7 in the vancomycin group, 20 in the gentamicin group, and 19 in the oATB group. Patients in the chronic dialysis program, with stage 4 and 5 chronic kidney disease, or concomitantly treated with another potentially nephrotoxic medication (cisplatin, colistin) or combination vancomycin/gentamicin were excluded. Initiation of acute intermittent hemodialysis or continuous RRT in study participants was not an indication for exclusion. The study is registered on ClinicalTrial.gov, ID: NCT04991376.

### Definition of sepsis

All patients had sepsis as defined by the Third International Consensus Definitions for Sepsis and Septic Shock (Sepsis-3) in 2016 [1]. The severity of organ dysfunction was assessed using the Sequential Organ Failure Assessment (SOFA) scoring system, with a total score of ≥2 representing organ dysfunction. The clinical presentation included the presence of inflammation (SIRS), a respiration rate > 22/min, change in mental status, and systolic blood pressure ≤ 100 mmHg (quickSOFA). In addition, other data were available for respiratory system status (PaO_2_/FiO_2_), coagulation status (platelet count), liver function (serum bilirubin), cardiovascular status (mean arterial blood pressure or need for vasopressors), the central nervous system (Glasgow Coma Scale Score), and the renal system (serum creatinine level and diuresis). Additional laboratory testing for IL-6 and PCT was routinely used in all patients to establish a diagnosis of sepsis and its severity.

### Definition of AKI

AKI was diagnosed and classified into three stages according to the recommendations of the 2012 KDIGO guidelines, based on the presence of increased S_crea_ concentration and/or decreased diuresis (KDIGO), or initiation of RRT [5]. Stage 1 AKI is defined as an increase in S_crea_ 1.5–1.9 times baseline or ≥ 0.3 mg/dL (≥26.5 mmol/L) and/or a decrease in urine output < 0.5 mL/kg/hour for 6–12 h. Stage 2 is defined as an increase in S_crea_ 2.0–2.9 times baseline and/or a decrease in urine output < 0.5 mL/kg/hour for ≥12 h. Stage 3 AKI has been defined as an increase in S_crea_ of 3.0 times baseline or ≥ 4 mg/dL (≥353.6 μmol/L) or initiation of RRT and/or a decrease in urine output < 0.3 mL/kg/hour for ≥24 h or anuria for ≥12 h [5]. We also used serum NGAL as an additional predictive marker [24].

### Determination of vancomycin and gentamicin serum concentrations

In patients treated with either vancomycin or gentamicin, we established the serum concentration of these antibiotics during treatment and adjusted dosage according to guidance from a clinical pharmacologist. At the University Hospital Ostrava, serum vancomycin concentrations were measured by liquid chromatography (LC)-tandem mass spectrometry (MS). A total of 50 μL of serum was precipitated using 20 μL 33% trichloroacetic acid, and 0.5 mol/L NH_4_OH was added to increase pH before analysis. For chromatographic analysis, we used a reversed-phase BEH C18.1.7 μm, 2.1 × 50 mm column maintained at 30 °C, with tobramycin as the internal standard. Mass detection was performed in positive electrospray mode. Performance characteristics of the method were as follows: linearity was between 98 and 105%, and the coefficients of variation were 2 to 7%. The results obtained with the LC-MS/MS method were correlated with those using a fluoroimmunoassay (Abbott AxSYM), as described previously [26]. Serum gentamicin concentrations were determined using a chemiluminiscence immunoassay method (Centaur, Siemens Healthcare Diagnostics Inc., Tarrytown, NY, USA) with an inter-assay coefficient of variation of 5%. Vancomycin and gentamicin doses were adjusted according to level, using the pharmacokinetic program MWPharm, version 3.30 (MEDIWARE, Groningen, the Netherlands/Prague, Czech Republic). In University Hospital Olomouc, a chemiluminiscence immunoassay method (Abbott Laboratories s.r.o., Diagnostic Division, Hadovka Office Park, Prague, Czech Republic) was used for determination of both vancomycin and gentamicin. For gentamicin, the inter-assay variation coefficient was ≤8%, and for vancomycin, it was ≤10%.

### Data and sample collection

Whole blood samples were taken on the first, fourth, and seventh days of antibiotic treatment. The first 24 samples were extracted in duplicate for screening of the selected miRNAs (see below). Concomitantly, the blood samples for NGAL determination were collected into 2.6 mL neutral tubes (S-Monovette® K3 EDTA, 2.6 mL, red, Sarstedt AG & Co. KG, Germany; or Vacuette® K3 EDTA 2 mL, violet, Greiner Bio-One GmbH, Germany) and centrifuged. After centrifugation, the plasma was aspirated, collected into cryotubes, and frozen at − 70 °C until use.

The blood samples for IL-6, PCT, and S_crea_ concomitantly with other standard care biochemical parameters also were collected (S-Monovette® serum-gel, 4.9 mL, brown, Sarstedt AG & Co. KG, Germany in University Hospital Ostrava; or Vacuette® serum-gel, 5.0 mL, red, Greiner Bio-One GmbH, Germany in University Hospital Olomouc) and analyzed immediately after centrifugation in both hospitals.

### Determination of NGAL, IL-6, PCT, and S_crea_

Serum concentrations of NGAL for all patients were determined in a certified laboratory of the Department of Clinical Biochemistry, University Hospital Ostrava, using the immunoturbidimetric method (Bioporto, AU 5820, Beckman Coulter, Inc., Brea, CA, USA). The inter-assay variation coefficient for NGAL determination was lower than 5%. The physiological range of plasma NGAL concentrations is 37 to 106 μg/L. Determination of IL-6, PCT, and S_crea_ was done separately in the certified laboratories of the cooperating hospitals.

The serum concentrations of IL-6 and PCT were determined at University Hospital Ostrava using the ADVIA Centaur immunoassay system (Siemens Healthcare Diagnostics Inc., Tarrytown, NY, USA) with coefficients of variation of 4.7 and 4.6%, respectively. At University Hospital Olomouc, electrochemiluminiscent immunoassays were used (IL-6 reagent kit, Cat. No. 05109442, Cobas 8000, Roche Diagnostics; Elecsys® BRAHMS PCT reagent kit Cat. No. 08828644, Cobas 8000, Roche Diagnostics, GmbH, Sandhofer Strasse 116, D-68305 Manheim, Germany). The physiological ranges are 0 to 4.4 ng/L for serum IL-6 and 0 to 0.5 μg/L for PCT. At University Hospital Ostrava, creatinine serum was assessed using the AU 5820 (Beckman Coulter, Inc., Brea, CA, USA) enzyme spectrophotometry method, and mass absorption spectrometry (Cobas 8000, CREP2, number 05168589 190, Roche Diagnostics GmbH, Sandhofer Strasse 116, D-68305 Manheim) was used at University Hospital Olomouc. The physiological ranges of serum creatinine are 45 to 90 μmol/L for women and 60 to 105 μmol/L for men.

### Determination and isolation of miRNAs

Whole 148 blood samples collected into PAXgene® Blood RNA tubes (762,165, PreAnalytix) from 53 patients were used for specific miRNA determination. Blood samples were collected into 2.5 mL tubes, stored at room temperature for no less then 2 h and up to 48 h, (Vacutainer® PAXgene, PreAnalytiX® GmbH, A Qiagen/BD Company, Switzerland), frozen at − 20 °C for 24 h, and then stored at − 80 °C until analysis. Body fluids provides much less miRNAs compared to cells or tissues therefore we used for our study whole blood collection. Values for 138 samples were included in the statistical analysis. Six patients did not have the required number of blood samples on day 4 or 7 for miRNA determination and were excluded from the statistical analysis.

Based on a literature search, we chose 20 miRNAs for their associations with sepsis-induced AKI (*hsa-miR-423-5p*, *hsa-miR-15a-5p*, *hsa-miR-4321-5p*, *hsa-miR-4270*, *hsa-miR-155-5p*, *hsa-miR-486-5p)* (blood) [20], AKI (*hsa-miR-320a*, *hsa-miR-142-3p* (blood/plasma) [21], *hsa-miR-21-5p* (blood and urine) [22]), vancomycin-induced nephrotoxicity (*hsa-miR-192-5p)* (HK-2 cells, renal cortical tissue) [16], *hsa-miR-301a-5p* (HK-2 cells, renal cortical tissue) [17]), sepsis (*hsa-miR-16-5p, hsa-miR-223-5p* (blood/plasma) [14], *hsa-miR-133a-3p* (blood/serum) [23]), and tubular injury and gentamicin-induced nephrotoxicity (*hsa-miR-320b*, *hsa-miR-203a-3p)* (urine) [19], *hsa-miR-218-1-3p*, *hsa-miR-489-3p, hsa-miR-138-5p* (urine) [18, 27]). Selected miRNA targets were examined by RT-qPCR method to determinate appropriate target miRNA for miREIA measurement and miRNA endogenous control.

### miRNA isolation

Isolation of target miRNAs was performed at the Institute of Laboratory Diagnostics, Department of Clinical Biochemistry, University Hospital of Ostrava, using the RNA isolation kit BioVendor Laboratory Medicine Corp., batch No. RIK001). The concentration of the target miRNA as measured using a microRNA enzymatic immunoassay (miREIA) can be affected by the efficiency of RNA isolation. For this reason, isolation efficiency was monitored by addition. In this case, the exogenous control used for normalization was *cel-miR-39-3p* from the kit (BioVendor – Laboratory Medicine Corp., batch No. RDM0000C), at a concentration of 10 nmol/L.

RNA purification was perfomed according manufacturer’s protocol. 2,5 mL of whole blood were collected into PAXgene® Blood RNA tube and stored frozen until the day of use. The frozen tubes were incubated 2 h at RT. Then centrifuged 12 min/ 4500 g. The supernatant was discarded and the sediment was dissolved with RNAse-free water and again centrifuged. The supernatant was again discarded and sediment homogenized with Qiazol (ID 79306, Qiagen). 160 μL of chloroform was added to each vial and mixtures were transfer to the new vials. Samples were incubated and then centrifuged 20 min/12000 g. The upper phase of the samples were transferred to a new collection microtube (2 mL), where “SPIKE in Control” sample (exogenous control *miR-39-3p*; 10 fmol. μL-1) and BB buffer were added. Mixture of sample and BB buffer is transferred into RNA isolation column. After several steps of washing, pure RNA including miRNA is eluted and ready for next analysis.

### RTqPCR screening

The first set for miRNA screening consisted of 24 isolate samples collected on days 1, 4, and 7 of treatment: six from two patients in the vancomycin group, nine from three patients in the gentamicin group, and nine from three patients in the oATB group. Selection of these samples was based on relevant biochemical characterization. Samples were sent for analysis to BioVendor (Laboratory Medicine Corp., Brno, Czech Republic). Quantification of the total RNA concentration of isolates was performed using a NanoDrop microvolume spectrophotometer (ND-2000, SN T463 NanoDrop Spectrophotometer, Thermo Fisher Scientific, Wilmington, MA, USA) (Table 1).

**Table 1:** Total RNA in 24 initial isolates

Sample number/days 1, 4, or 7 and treatment	Total RNA (ng/μL)	Initial concentration to RT (ng/μL)	Dilution factor (×)
1/1 gentamicin	173.3	5	35
1/4	235.9	10	24
1/7	459.3	10	46
2/1 gentamicin	78.4	5	16
2/4	72.3	5	14
2/7	72.4	5	14
3/1 vancomycin	166.3	5	33
3/4	167.6	5	34
3/7	230.3	10	23
4/1 vancomycin	170.3	5	34
4/4	134.1	5	27
4/7	175	5	35
5/1 other ATB	203.2	10	20
5/4	1084.5	10	108
5/7	1459.5	10	146
6/1 other ATB	135.1	5	27
6/4	152.6	5	31
6/7	109.8	5	22
7/1 gentamicin	146.5	5	29
7/4	263.3	10	26
7/7	435.8	10	44
8/1 other ATB	192.8	10	19
8/4	222.8	10	22
8/7	360	10	36

Abbreviations: *ATB* antibiotic, *RNA* ribonucleic acid, *RT* reverse transcription

A screening of specific miRNAs was performed on these patient isolates using quantitative reverse transcription PCR (MiRXES ID3AL™ miRNA qPCR).

The MiRXES microRNA Assays are quantitative RT-PCR assays and are designed to detect and accurately quantify microRNAs. The principle of the MiRXES microRNA Assays is based on the combination of unique miRNA specific RT primer and nested qPCR primer pairs and it enables to efficiently discriminate highly homologous miRNA family members with single nucleotide difference. The ID3EAL™ miRNA qPCR Assays yields class leading sensitivity compared to other miRNA detection systems. MiRXES MicroRNA Assays include reverse transcription and real-time PCR two steps only. RNA is reverse transcribed to cDNA using a gene specific primer.

Prior to reverse transcription, the concentration of RNA isolates was verified and 5 (5–10) ng/μl of total RNA was combined for total reaction volume in 20 μl with: 5 μl ID3EAL RT Buffer (4x), 1 μl of ID3EAL RT Primer I-Plex I (20x), 1 μl of ID3EAL RT Primer I-Plex N (20x), 1 μl ID3EAL Reverse Transcriptase (20x) and remainder of the reaction was made up of nuclease- free water. All components can be found in the MiRXES ID3EAL™ cDNA Synthesis System and MiRXES ID3EAL™ Individual miRNA RT Primer 1-plex, (P/N 1103103, 1,103,113, BioVendor – Laboratory medicine a.s., Brno, Czech Republic). The RT reactions were incubated on a CFX96 TOUCH REAL-TIME PCR detection system thermocycler: 42 °C/30 m followed with heat inactivation at 95 °C/5 m, 4 °C/hold. Each individual RT reaction has a unique ID3EAL™ miRNA qPCR Master Mix and ID3EAL™ miRNA qPCR Assays designed for it (P/N 1104101, 1,104,204, 1,104,202, BioVendor – Laboratory medicine a.s., Brno, Czech Republic). The dilution of cDNA obtained by reverse transcription was 10 times in nuclease free water. PCR reactions were assembled for 20 μl reaction volume (recommended if using 96-well plates), combine 10 μl 2x ID3EAL qPCR MasterMix, 5 μl diluted cDNA, 2 μl 10x ID3EAL miRNA qPCR assays, and 3 μl nuclease-free water. Real-time PCR amplification was performed incubation in a 96-well plate at 95 °C for 10 min, 40 °C for 5 min. and followed by 40 cycles of 95 °C for 10 s and 60 °C for 30 s. The real-time PCR reactions was performed in triplicate and use the averages, discarding any outlier (> 2 standard deviations), to perform subsequent analyses. Inappropriate miRNA targets were evaluated due to miRNAs sequence specificity and to the poor yield of the target.

From these results, we identified four miRNAs with the most significant expression (lower mean threshold cycles) in terms of specificity and yield of selected miRNA sequences (Table 2).

**Table 2:** RT-qPCR analysis of miRNAs of the first 24 isolates

hsa-miRNA	*133a-3p*	*15a-5p*	*16-5p*	*4270*	*218–1-3p*	*320b*	*203a-3p*	*138-3p*	*489-3p*	*301a-5p*	*423-5p*	*4321*	*192-5p*
¢Ct	35.2	**18.8**	34.6	30.8	33.6	**19.1**	32.4	36.2	36.1	29.7	**18.3**	39.1	**19.9**
max Cq	35.8	22.3	37.1	31.5	35.7	20.8	34.1	38.9	37.6	31.3	19.6	40.4	22.8
min Cq	33.8	14.6	31.2	29.1	31.9	17.0	30.4	33.6	33.6	27.9	17.1	36.7	18.4

Abbreviations: *¢Ct* mean threshold cycles qRT analysis, *Cq* quantification cycle, *hsa-miRNA* human microRNA, *RT-qPCR* quantitative reverse transcription PCR

After this calculation, we performed a biological normalization of the concentrations of the target miRNAs against an endogenous control (*hsa-miR-486-5p*). A geometrical mean was calculated from the achieved *hsa-miR-486-5p* concentrations. By taking the ratio of the mean and the normalization control concentration of *hsa-miR-486-5p*, we obtained the normalization factors for each sample. All concentrations of the target miRNAs were multiplied by these factors. The software geNorm was used for analysis of potentially normalized genes (Table 3).

**Table 3:** Endogenous control–normalized genes

Gene	Expression viability value
*hsa-miR-155-5p*	0.073
*hsa-miR-320a*	0.077
*hsa-miR-486-5p*	0.088
*cel-miR-39-3p*	0.081
*hsa-miR-223-5p*	0.099
*hsa-miR-142-3p*	0.111

For the final analysis, however, *miR-320b* was changed to endogenous *miR-155-5p*. Detailed analysis of initial data from screening have been show more significant relevance of *miR-155-5p* for determined samples of this study.

### miREA

Based on the preliminary screening, we chose four miRNAs kit *hsa-miR-15a-5p*, (batch No. RDM0007H); kit *hsa-miR-155-5p*, a change from the initially predicted *miR-320b*, (batch No. RDM0017H); kit *hsa-miR-192-5p*, (batch No. RDM0035H); kit *hsa-miR-423-5p*, (batch No. RDM0033H), together with one endogenous control kit *hsa-miR-486-5p* [20], (batch No. RDM0022H); all kits by BioVendor – Laboratory Medicine Corp. These specific miRNAs were gradually determined by the miREIA (BioVendor – Laboratory Medicine Corp., Brno, Czech Republic).

MiREIA is a novel, immunoassay-based method of miRNA quantification. The biotin-labeled specific DNA oligonucleotide is hybridized with isolated miRNA from the blood sample. The DNA/RNA heterohybrids are then transferred onto a stationary solid phase coated with a specific monoclonal antibody. The washed solid phase is then incubated with streptavidin–horseradish peroxidase conjugate and visualized using a chromogenic substrate (tetramethylbenzidine). The absorbance of the solution is determined at a wavelength of 450 nm and corresponds to the concentration of specific miRNA species present in the blood sample (BioVendor – Laboratory Medicine Corp., Brno, Czech Republic). With the measured concentration results for each selected miRNA, we first performed a so-called technical normalization of the concentrations. For this calculation, an efficiency ratio of isolation for each sample was determined, based on division of an expected concentration of the exogenous control (*cel-miR-39-3p*) by the concentration yielded from measuring the target miRNA. The concentration of each target miRNA then was multiplied by this ratio.

### Statistical analysis

The Shapiro – Wilk test was used to test the normality of the data. Because no data had a normal distribution, we applied nonparametric tests for statistical analysis. We used the Mann–Whitney U test to compare two groups, and for comparison of three groups, we used the Kruskal–Wallis test. For post hoc adjustment for multiple comparisons, we applied Dunn’s multiple comparison test, and we used the Friedman test to evaluate significance across more than two groups of dependent data. For multiple comparisons, we used the Wilcoxon signed-rank test.

To evaluate correlations between miRNA scores and NGAL, PCT, IL-6, and S_crea_, and other biochemical or clinical parameters respectively, we used the Spearman correlation coefficient. Multivariate analysis was done by a principal component analysis, with a biplot given as a graphical interpretation of the multidimensional data. Simple logistic regression with the log-likelihood ratio test was used to analyze the association between the miRNAs and AKI stage. Significance was set at alpha = 0.05, and statistical analyses and graphic representations were done using R-project [28], version 4.0.3 (2020-10-10) with the dunn.test, Paired Data, ggplot2, factoextra, corrplot, and ggpubr packages, and IBM SPSS Statistics 27 and GraphPad Prism version 9.0.1 (128).

## Results

### Clinical, demographic and biochemical characteristics of patients

The basic clinical and demographic characteristics of the patients are summarized in Table 4.

**Table 4:** Clinical and demographic characteristics of the patients

	Vancomycin group	Gentamicin group	oATB group	P
N (%)	7 (15)	20 (43)	19 (41)	
Age (years)	65 (47–76)	59 (47–72)	65 (63–68)	0.785
Male n (%)	4 (57)	13 (65)	13 (68)	0.866
BMI (kg/m^2^)	26.2 (23.3–27.8)	28.4 (24.0–32.5)	26.3 (24.7–30.9)	0.620
SOFA score (%)	10 (7–14) + 1 QuickSOFA	8 (5–12)	7 (3–10)	0.406
AKI stage at baseline/end of study, all n (%)				
1	1 (14) / 1 (14)	5 (25) / 3 (15)	4 (21) / 3 (16)	0.836/0.995
2	N	5 (25) / 1 (20)	6 (32) / 0	0.243/0.515
3	2 (29) / 2 (29)	2 (10) /5 (5)	6 (32) / 9 (47)	0.236/0.318
No AKI	4 (57) / 4 (57)	8 (40) / 11 (55)	3 (15) / 7 (37)	0.088/0.455
RRT initiation, n (%)	2 (2)	5 (25)	7 (37)	0.719
Use of vasopressors, n (%)	6 (86)	15 (75)	11 (58)	0.307
Type of vasopressor, n (%)				
Norepinephrine	6 (100)	14 (93)	8 (73)	0.172
Dobutamine	N	N	1 (9)	0.484
Dopamine	N	N	1 (9)	0.484
Norepinephrine + Dobutamine	N	1 (7)	1 (9)	0.416
Dose of vasopressors (μg/kg/min)				
Norepinephrine	0.18 (0.14–0.23)	0.21 (0.17–0.29)	0.32 (0.22–0.45)	0.104
Dobutamine	N	N	1.79 (1.19–2.38)	0.333
Dopamine	N	N	24.41 (19.41–29.41)	0.333
Norepinephrine / Dobutamine	N / N	0.14 (0.001–0.28) / 1.04 (0.69–1.39)	0.24 (0.002–0.48) / 11.63 (0.16–23.10)	0.467/0.600
Duration of vasopressors therapy (days)	10 (6–12)	6 (2–9)	6 (3–13)	0.256
ICU hospitalized patients, n (%)	6 (86)	18 (90)	15 (79)	0.629
ICU length of stay (days)	18 (11–38)	13 (9–23)	12 (10–22)	0.585
Intermediate care hospitalized patients, n (%)	1 (14)	2 (10)	4 (21)	0.629
Intermediate care length of stay (days)	12	9 (8–10)	16 (10–24)	0.438
Total length of hospitalization (days)	31 (12–52)	25 (15–35)	17 (12–24)	0.162
Diagnoses, all n (%)				
Urogenital infection	2 (29)	4 (20)	5 (26)	0.856
Pneumonia				
- Nosocomial	1 (14)	2 (10)	2 (11)	0.950
- Community acquired	N	N	2 (11)	0.226
Sepsis in severe trauma	1 (14)	4 (20)	N	0.127
Peritonitis	1 (14)	6 (30)	1 (5)	0.122
Mediastinitis	1 (14)	1 (5)	N	0.280
Liver abscess	1 (14)	N	N	0.058
Acute cholecystitis	N	N	1 (5)	0.484
Acute pancreatitis	N	N	1 (5)	0.484
Infectious gastroenteritis	N	N	1 (5)	0.484
Abscess or phlegmon	N	2 (10)	3 (16)	0.511
Erysipelas	N	N	1 (5)	0.484
Sepsis of unknown origin	N	1 (5)	2 (11)	0.587
Clinical outcome survived/died, n (%)	6 (86) / 1 (14)	18 (90) / 2 (10)	15 (79) / 4 (21)	0.629

Abbreviations: *AKI* acute kidney injury, *BMI* body mass index, *ICU* intensive care unit, *N* none, *oATB group* other ATB group, *RRT* renal replacement therapy, *SOFA* Sequential Organ Failure Assessment score

Quantitative variables are presented as medians and interquartile ranges. Qualitative variables are presented as counts and percentages. *P* values are from Kruskal–Wallis tests for quantitative variables and Pearson’s χ2 tests for qualitative variables

There was no statistical difference among the three groups in age, sex, body mass index, SOFA score or AKI stage at sepsis diagnosis and treatment initiation. Furthermore, there was no statistical difference in use of vasopressors, type, median of dose and duration of their administration, ICU length of stay or clinical outcome among the defined groups of patients. Antimicrobial therapy in all groups was initiated empirically according to supposed bacterial agent or according to type of clinical diagnosis, with microbiological results as follows: in the vancomycin group, the cause was *Enterococcus* sp. in 57% of cases, *Staphylococcus epidermidis* together with *Klebsiella pneumoniae* in 29%, and *Staphylococcus hominis* in 14%. Fungal superinfection caused by *Candida* sp. was detected in 43%. In the gentamicin group, the cause was *Escherichia coli* in 30% of cases, *Staphylococcus* sp. in 20%, *Klebsiella pneumoniae* in 15%, and *Enterobacter* sp., *Pseudomonas aeruginosa*, *Burkholderia* sp., or *Providencia rettgeri* in 5%. and *Candida* sp. as superinfection in 20%. No bacterial, fungal or viral agent was established in 15% of these cases. Among those treated with oATBs, the etiological bacterial agent was *Escherichia coli* in 21%, *Klebsiella* sp. and *Staphylococcus hominis* in 11%, and *Enterobacter* sp., *Salmonella enteritidis*, *Burkholderia* sp., *Pseudomonas aeruginosa*, and *Acinetobacter* sp. in 5% and fungal superinfection caused by *Candida* sp. in 16% and *Aspergillus fumigatus* in 11%. In 32% of cases in the oATB group, no bacterial, viral or fungal agent was established during the time of observation. The combination or change of antibiotic agents according to obtained microbiological results was adjusted according to patient’s need.

Vancomycin therapy was usually administered in combination with antimycotics (fluconazole or anidulafungin) and/or metronidazole. Beta-lactams, metronidazole, cephalosporins, and penicillins were added to the combination therapy with gentamicin. In the oATB group, we used beta-lactams, cephalosporins, sulfonamides, macrolides, quinolones, antimycotics (fluconazole or anidulafungin), or their combination, according to identified or inferred agent. The duration of the antimicrobial treatment depended on patient laboratory and clinical status. In the vancomycin group, the median treatment delay was 10 (interquartile range, 6–13) days, with a median first trough vancomycin serum concentration of 10.1 (6.5–14.0) mg/L. The median vancomycin dose was 23.5 (13.2–54.5) mg/kg/24 h. In the gentamicin group, the median treatment time was 7 (6–9) days, with a median maximum serum concentration of gentamicin of 14.6 (11.1–17.4) mg/L and median minimum serum concentration of 0.7 (0.3–0.9) mg/L, below the threshold of nephrotoxicity (≥2 mg/L). The median gentamicin loading dose was 3.5 (3.0–4.0) mg/kg/24 h. In the oATB group, treatment duration depended on the type of antibiotic used and the patient’s clinical and laboratory status, with a median of 11 (8–14) days.

The median concentrations of the inflammation (PCT, IL-6) and renal injury (S_crea_ and NGAL) markers were not statistically significantly different among the three groups on days 1, 4, or 7 (Table 5). However, there was observed statistically significant difference among the defined groups between medians of serum urea (nonspecific marker of renal injury, metabolic disorders and fluid balance) (*p* = 0.049) and C-reactive protein (CRP) (nonspecific marker of inflammation) concentration (*p* = 0.013), only on day 1.

**Table 5:** Biochemical parameters of sepsis and renal injury among the patients

Parameter	Day	Vancomycin	IQR	Gentamicin	IQR	Other ATBs	IQR	P
NGAL (μg/L)	1	240	120–1150	290	136–937	468	210–775	0.558
NGAL (μg/L)	4	170	100–520	217	105–351	438	292–488	0.105
NGAL (μg/L)	7	130	75–430	157	125–476	358	200–583	0.172
S_crea_ (μmol/L)	1	71	59–165	116	87–166	156	131–239	0.063
S_crea_ (μmol/L)	4	59	45–139	85	68–98	129	75–226	0.064
S_crea_ (μmol/L)	7	62	42–225	78	57–108	101	66–160	0.378
IL-6 (ng/L)	1	51	31–109	310	61–5500	176	73–1073	0.107
IL-6 (ng/L)	4	34	13–109	47	19–185	55	25–108	0.598
IL-6 (ng/L)	7	23	11–92	27	12–152	66	20–289	0.551
PCT (μg/L)	1	0.5	0.2–9.8	7.9	0.7–22.7	2.5	1.5–5.9	0.162
PCT (μg/L)	4	0.3	0.1–2.7	1.5	0.3–1.8	1.5	0.5–5.1	0.297
PCT (μg/L)	7	0.2	0.0–3.0	0.7	0.1–1.8	0.7	0.2–1.3	0.530

Abbreviations: *IL-6* interleukin 6, *IQR* interquartile range, *NGAL* neutrophil gelatinase–associated lipocalin, *PCT* procalcitonin, *S*_*crea*_ serum creatinine. *P* values derived from the Kruskal–Wallis test. Data are presented as medians and interquartile ranges. IL-6 on day 7 in the gentamicin group is presented for 19 patients; day 7 data for IL-6, S_crea_, and PCT are presented for 18 patients in the ATB group

### Selected miRNAs expression

**Fig. 1:**
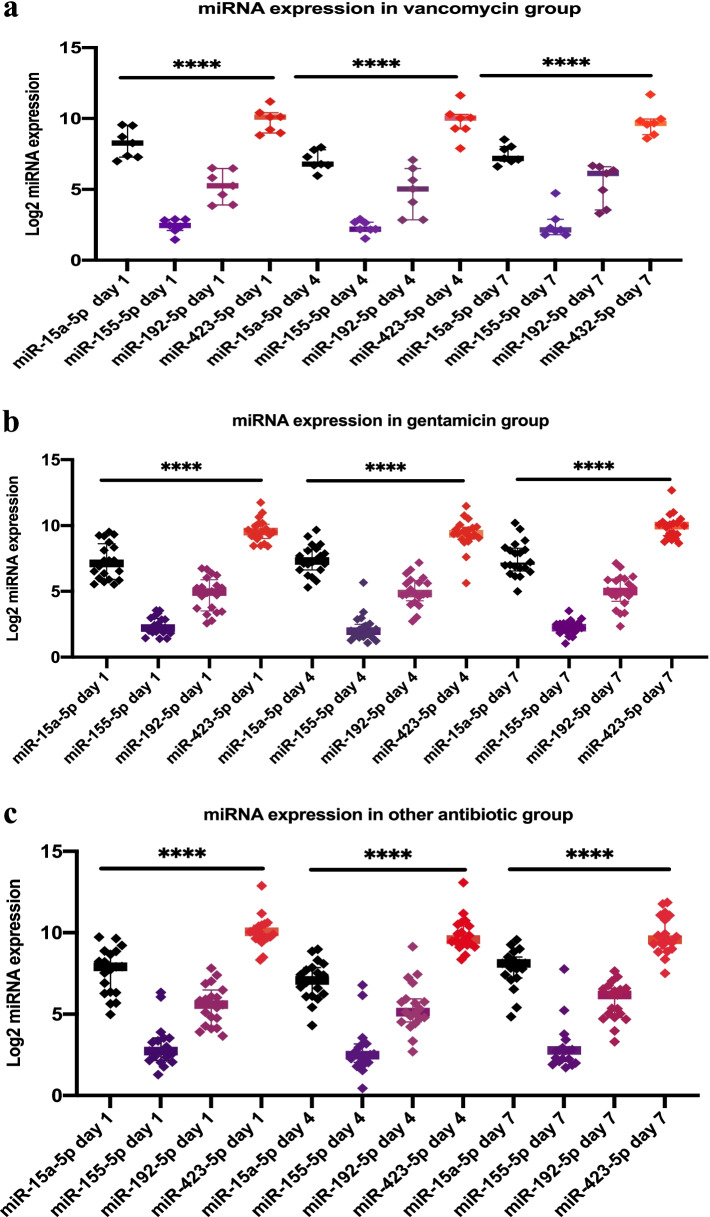
Similar pattern of individual expression of four miRNAs in each treatment group on days 1, 4, and 7 of treatment. **a** Vancomycin group, **b** gentamicin group, and **c** other ATB group. The figures show the log 2-transformed label-free quantification of the four investigated miRNAs during the treatment period. *P* values are from Kruskal–Wallis test, and **** indicates *p* < 0.001 between the miRNAs themselves. Post-hoc analysis was performed using Dunn’s method with Bonferroni correction. Each investigated miRNA presented a similar respective pattern of expression across all groups

We found no difference in median expression values for the four analyzed miRNAs among the patient groups on days 1, 4, and 7. However, the groups all showed a similar pattern of expression for each miRNA, which differed among the miRNAs, through the 7-day period (Fig. 1).

**Fig. 2:**
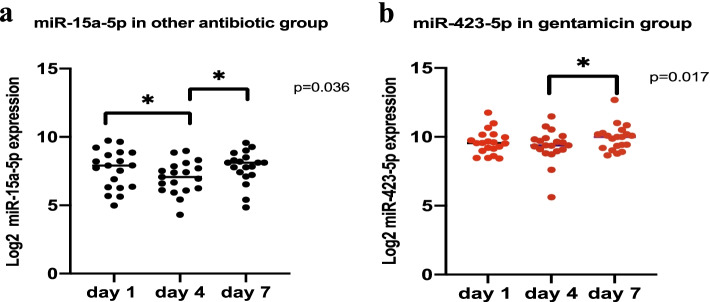
Time-dependent change in expression of *miR-15-5p* and *miR-423-5p* in the other-antibiotic group and the gentamicin group (vancomycin not shown). Log 2–transformed label-free quantification of differential expression of two miRNAs. **a** *miR-15-5p* in the other-ATB group. The figure shows a significant change in expression of *miR-15a-5p* between days 1 and 4 and days 4 and 7. **b** *miR-423-5p* in the gentamicin group, in which a statistically significant change was detected between days 4 and 7. Data are presented as medians and interquartile ranges. *P* values are the result of the Friedman test and post-hoc Wilcoxon test

We also identified a statistically significant change in serum concentrations and up- and down expression for *miR-15a-5p* in the oATB group and *miR-423-5p* in the gentamicin group (Fig. 2).

We found no significant differences in NGAL concentrations among the groups or in IL-6 in the vancomycin group at 7 days. However, the median NGAL serum concentration in all groups was elevated throughout the study, above the threshold physiological concentration of 106 μg/L.

### miRNAs expression and relationship with biochemical parameters

**Fig. 3:**
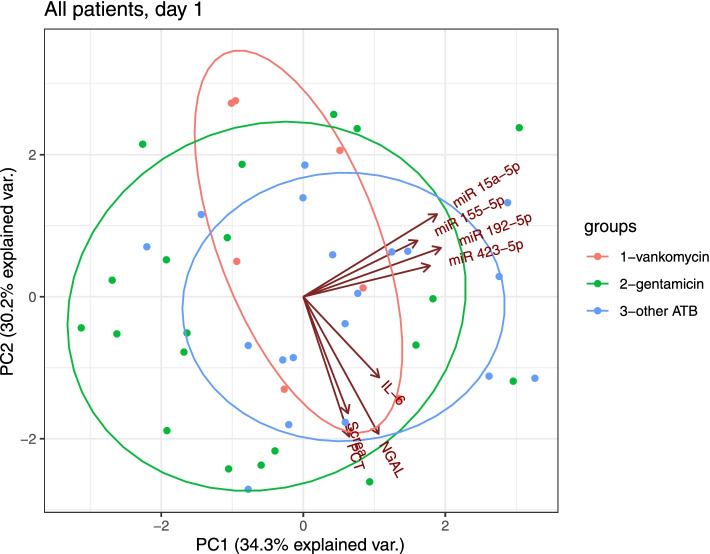
Principal component analysis of the relationship between miRNAs and biochemical variables on study day 1. Principal component analysis biplot shows log 2–transformed label-free quantification of the concentrations of variables and the difference between miRNA expression and renal injury or inflammation markers on day 1 in all patients, regardless of subsequent treatment. No relationship was identified between miRNAs and biochemical variables

The principal component analysis plot of the investigated parameters (miRNAs, NGAL, PCT, IL-6, and S_crea_) on study day 1 is shown in Fig. 3. No miRNA showed a relationship with renal injury or inflammation markers. We also found no correlation (Spearman) between the investigated miRNAs and biochemical markers. Furthermore, no correlation on day 1 has been observed between miRNAs and additional biochemical parameters as urea, CRP, albumin, lactate or bilirubin (*p* > 0.05).

Statistically significant positive correlations between miRNAs and biochemical variables during the study are presented in Table 6. We observed no correlations between any miRNA and biochemical markers in the gentamicin group (not shown) and no correlations between miRNAs and clinical parameters as SOFA score, patient’s weight or BMI in any group (not shown).

**Table 6:** Correlations between miRNAs and biochemical markers

Group	Parameter	Spearman r	hsa-miR-155-5p	hsa- miR-192-5p	hsa-miR-423-5p
Day 4					
Vancomycin	**S**_**crea**_	r_s_	0.786	**–**	**–**
		P	**0.048**	**–**	**–**
	**NGAL**	r_s_	0.821	**–**	**–**
		P	**0.034**	**–**	**–**
oATB	**IL-6**	r_s_	**–**	0.642	–
		P	**–**	**0.003**	**–**
all patients	**NGAL**	r_s_	0.351	0.339	0.419
		P	**0.017**	**0.021**	**0.004**
	**IL-6**	r_s_	0.328	**–**	**–**
		P	**0.026**	**–**	**–**
Day 7					
Vancomycin	**S**_**crea**_	r_s_	–	0.821	–
		P	–	**0.034**	–
	**NGAL**	r_s_	–	0.929	–
		P	–	**0.007**	–
	**PCT**	r_s_	–	0.847	–
		P	–	**0.025**	–
oATB	**PCT**	r_s_	–	0.558	0.643
		P	–	**0.016**	**0.004**
	**IL-6**	r_s_	–	0.598	0.719
		P	–	**0.009**	**< 0.001**
all patients	**PCT**	r_s_	0.413	0.413	0.550
		P	**0.005**	**0.005**	**< 0.001**
	**IL-6**	r_s_	0.357	–	0.491
		P	**0.017**	**–**	**< 0.001**
	**NGAL**	r_s_	0.523	**–**	0,421
		P	**< 0.001**	**–**	**0.004**
	**S**_**crea**_	r_s_	0.419	**–**	0.313
		P	**0.004**	**–**	**0.036**

Abbreviations: *IL-6* interleukin 6, *NGAL* neutrophil gelatinase–associated lipocalin, *PCT* procalcitonin, *r*_*s*_ Spearman correlation coefficient r, *S*_*crea*_ serum creatinine. P per Spearman correlation coefficient

No correlation in gentamicin group was found - not shown

A significant change in AKI stage from days 1 to 7 was seen for the overall patient population (*p* = 0.009), and separately in the oATB group (*p* = 0.039). In the gentamicin group on day 1, however, we found a significant relationship between AKI stage and serum NGAL concentration (*p* = 0.039), which we also found in the oATB group on day 7 (*p* = 0.030). In all patients together we found on day 4 positive correlation between NGAL and *miR-155-5p* (*p* = 0.017), *miR-192-5p* (*p* = 0.021) and *miR-423-5p* (*p* = 0.004) as well as between IL-6 and *miR 155-5p* (0.026). Furthermore, on day 7 two miRNAs: *miR-155-5p* and *miR-423-5p* positively correlated with all biochemical markers and *miR-192-5p* positively correlated with PCT (*p* = 0.005) (Table 6).

**Fig. 4:**
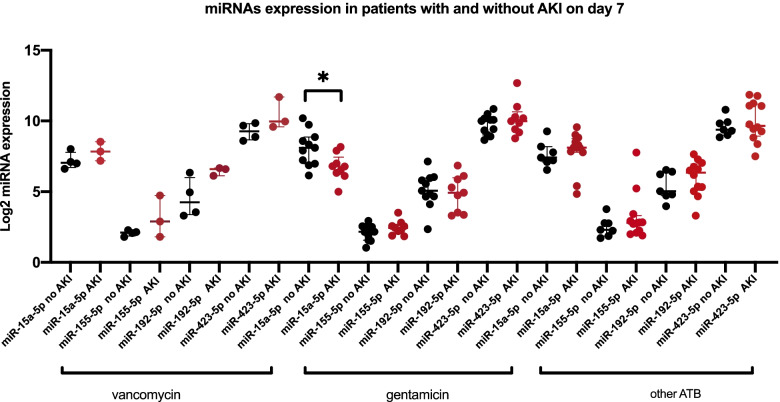
miRNA expression in patients with and without acute kidney injury (AKI) on day 7. Log 2–transformed label-free quantification of differential expression of miRNAs in patients with and without acute kidney injury in all three groups (vancomycin, gentamicin, other ATB) on day 7 is presented on the figure. The statistically significant difference between patients with and without acute kidney injury is observed in *miR-15a-5p* in gentamicin group. * - *p* value of Mann–Whitney U test (*p* = 0.013)

None of the investigated miRNAs showed any statistically significant association with AKI stage on day 1, but *miR-15a-5p* in the gentamicin group showed a statistically significant difference between patients with and without AKI on day 7 (Table 7 and Fig. 4).

**Table 7:** Concentrations of miRNAs in patients with and without acute kidney injury on day 7

Group	miRNA	No AKI		AKI		
		Median	IQR	Median	IQR	P
Vancomycin	*hsa-miR-15a-5p (amol/L)*	132.7	105.9–228.2	228.4	144.9–368.3	0.229
	*hsa-miR-155-5p (amol/L)*	4.3	3.7–4.7	7.4	3.5–26.6	0.400
	*hsa-miR-192-5p (amol/L)*	21.4	10.4–68.6	96.8	69.9–101.8	0.114
	*hsa-miR-423-5p (amol/L)*	640.4	407.5–895.0	1004.0	768.5–3313.0	0.229
Gentamicin	*hsa-miR-15a-5p (amol/L)*	274.7	125.5–467.0	109.6	75.1–182.6	**0.013**
	*hsa-miR-155-5p (amol/L)*	4.5	3.0–5.8	5.5	4.2–6.5	0.182
	*hsa-miR-192-5p (amol/L)*	33.4	25.1–62.7	30.4	10.9–63.9	0.824
	*hsa-miR-423-5p (amol/L)*	1024.0	517.2–1156.0	1016.0	634.9–1651	0.552
oATB	*hsa-miR-15a-5p (amol/L)*	171.2	137.4–292.0	277.8	222.0–432.6	0.299
	*hsa-miR-155-5p (amol/L)*	4.9	3.7–6.9	6.9	4.4–10.0	0.270
	*hsa-miR-192-5p (amol/L)*	32.8	26.4–85.3	81.9	34.6–125.6	0.227
	*hsa-miR-423-5p (amol/L)*	668.7	513.3–973.1	812.0	493.3–2358.0	0.592

Abbreviations: *AKI* acute kidney injury, *IQR* interquartile range, *miRNA* microRNA; *p* values per Mann–Whitney U test

**Fig. 5:**
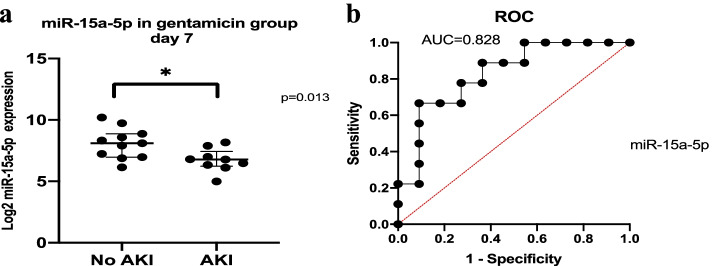
*miR-15a-5p* may be a biomarker for acute kidney injury (AKI) in patients with sepsis treated by gentamicin. Data are presented as log 2–transformed label-free quantification of miRNA serum concentrations and expression. **a** Serum concentrations of *miR-15a-5p* in patients with AKI versus non-AKI in the gentamicin group; *p* values are from the Mann–Whitney U test. **b** Relative receiver operating characteristic (ROC) curve with the area under the curve obtained from by simple logistic regression; ** log-likelihood ratio test, *p* = 0.008

Simple logistic regression with a log-likelihood ratio test was used to analyze the association of miRNAs with AKI presence and showed a significant influence of *miR-15a-5p* on AKI presence in the gentamicin group (*p* = 0.008) (Fig. 5). The receiver operating characteristic curve for *miR-15a-5p* in predicting AKI presence is shown in Fig. 5 and suggests that circulating *miR-15a-5p* could be a biomarker for AKI patients with sepsis treated with gentamicin.

Based on simple logistic regression, using the *miR-15a-5p* concentration, we postulated a probability equation for AKI in the gentamicin group. The point estimates and confidence intervals of the simple logistic regression were equal to 1.753 (− 0.076 to 4.121) for parameter β_0_ and − 0.010 (− 0.024 to − 0.002) for parameter β_1_.

The odds ratios with confidence intervals were 5.770 (0.927 to 61.640) for parameter β_0_ and 0.990 (0.977 to 0.998) for parameter β_1._ The AUC was 0.828. The predicted probability π of AKI is given by the formula:

$$\uppi =\frac{\mathit{\exp}\ \left(1.753-0.010\ \mathrm{x}\ miR\ 15a-5p\right)}{1+\exp\ \left(1.753-0.010\ \mathrm{x}\ miR\ 15a-5p\right)}$$

where *π* is a point estimate of probability that AKI will be equal to 1, which means AKI will be present.

## Discussion

**Fig. 6:**
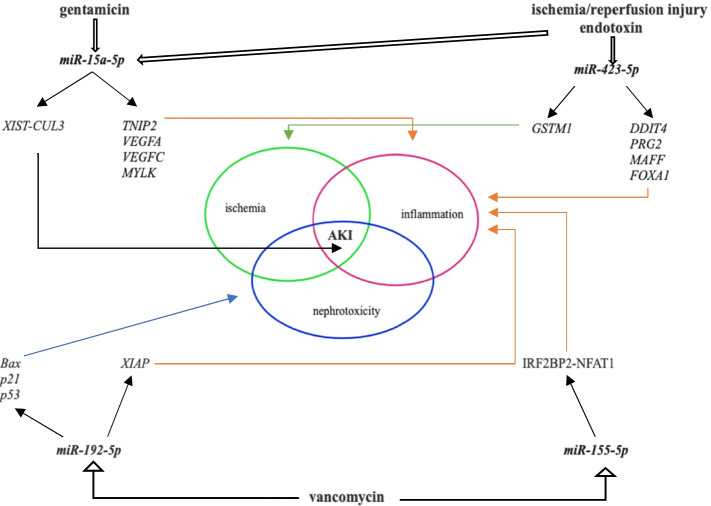
Simplified graphical presented associations between sepsis, nephrotoxic antibiotics, investigated miRNAs and their target regulatory genes in sepsis induced acute kidney injury. On the figure are presented simplified associations between gentamicin, vancomycin, endotoxin, ischemia/reperfusion injury and *miR-15a-5p*, *miR-423-5p*, *miR-192-5p and miR-155-5p* with their previously established target genes. Acute kidney injury in sepsis is a complex process involving combination of risk factors as ischemia, inflammation and possible nephrotoxicity. Abbreviations: AKI – acute kidney injury, Bax – B-cell lymphoma 2 Asociated X, Apoptosis Regulator, DDIT4 – DNA damage–inducible transcript 4, FOXA1 – forkhead box protein A1, GSTM1 – glutathione-S-transferase M1, IFR2BP2-NFAT1 – interferon regulatory factor 2 binding protein 2*–*nuclear factor of activated T cells 1, MAFF – MAF basic leucine zipper transcription factor F, miR – micro RNA, MYLK – myosin light chain kinase, PRG2 – proteoglycan 2, pro eosinophil, major basic protein, p21– p21 gene, p53 – p53 gene, TNIP2 –*TNFAIP3* interacting protein 2 gene, VEGFA – vascular endothelial growth factor A gene, VEGFC – vascular endothelial growth factor C gene, XIAP – X-linked inhibitor of apoptosis, XIST-CUL3 – X inactive specific transcript - cullin 3 gene

In this prospective clinical study, from among 20 candidates selected from previously published studies, we identified four circulating miRNAs — *miR-15a-5p*, *miR-155-5p*, *miR-192-5p*, and *miR-423-5p* — as relevant in critically ill patients with sepsis. In three groups of patients categorized by antibiotic treatment, we evaluated concentration changes in these circulating miRNAs during 7 treatment days. Two groups were treated with nephrotoxic agents (vancomycin or gentamicin), and the third group was treated with oATBs. The four circulating miRNAs each respectively showed a similar expression pattern during the 7 days of observation, regardless of antimicrobial therapy (vancomycin/gentamicin/oATB) (Fig. 1). In addition, they showed no significant relationship with renal (S_crea_, NGAL) and inflammatory markers (IL-6, PCT) on day 1 of the study period (Fig. 3). Furthermore, no correlation on day 1 has been observed between miRNAs and additional biochemical parameters as urea, C-reactive protein, albumin, lactate or bilirubin. Simplified graphical association between investigated miRNAs and their target genes/pathways, nephrotoxic antibiotics, sepsis and acute renal damage is presented on Fig. 6.

### miR-423-5p

Among the four miRNAs, *miR-423-5p* was the most expressed in all patients. Human *miR-423-5p* has up to 700 predicted target genes [27]. Of these, genes encoding DNA damage–inducible transcript 4 (*DDIT4*), proteoglycan 2, pro eosinophil, major basic protein (*PRG2*), and MAF basic leucine zipper transcription factor F (*MAFF*) have been recently determined to be upregulated in pediatric patients suffering from septic shock [29]. *DDIT4* is induced under cellular stress conditions and regulates mTOR activity. Furthermore, *DDIT4* is upregulated in response to hypoxia-inducible factor 1, regulates the generation of reactive oxygen species, and is a p53 target induced by DNA damage [30]. *PRG2* encodes a protein that may be involved in antiparasitic defense mechanisms as a cytotoxin and that can have potent antimicrobial activity against fungi and Gram-positive and -negative bacteria [31]. *MAFF*, among its the other functions, may be involved in the cellular stress response [31].

In the gentamicin group, *miR-423-5p* showed a time-dependent change in expression from day 4 to day 7 (Fig. 2). In the oATB group, circulating *miR-423-5p* levels positively correlated with the inflammatory markers IL-6 and PCT and on day 7. Furthermore, in all patients together *miR-423-5p* positively correlated with NGAL (day 4) and with all observed biochemical markers (day 7) (Table 6). *miR-423-5p* plays a substantial role in pathophysiological processes related to ischemia and hypoperfusion. Experimentally in an ischemia-reperfusion (I/R) injury model, *miR-423-5p* was upregulated with induced endoplasmic reticulum stress and reactive oxidative stress by inhibiting glutathione-S-transferase M1 (GSTM1), leading to apoptosis of proximal tubular cells [32] (Fig. 6). Significant upregulation of *miR-423-5p* has been reported in patients in the early phase of myocardial infarction and with its normalization in 6 h [33]. In a study of 179 patients suffering from cardiogenic shock, overexpression of *miR-423-5p* was associated with organ ischemia, presenting as a higher lactate concentration and organ injury with a lower cardiac index, and with increased 90-day mortality [34]. In one clinical study of 98 patients with acute heart failure, Bruno et al. found that increased S_crea_ corresponded to lower circulating *miR-423-5p* concentrations, which was not associated with serum NGAL [35]. In all of the patients in the current study, the median NGAL serum concentration stayed above the physiological range of 37–106 μg/L (Table 5), and circulating *miR-423-5p* positively correlated with NGAL serum concentrations in all patients together on day 4 and 7 and with S_crea_ only on day 7 (Table 6). Furthermore, according to Martensson et al., plasma NGAL rises with sepsis and septic shock and should be used with caution as a marker of AKI in ICU patients with septic shock [36]. In another clinical study of patients with membranous glomerulopathy, *miR-423-5p* was upregulated, in addition to nine other miRNAs, and positively correlated with proteinuria, suggesting an association with worsening glomerular damage. Upregulation of *miR-423-5p* also has been observed in renal biopsy tissues from patients with membranous glomerulopathy, accompanied by a decrease in IL-6, in contrast with our current observations [37]. In an experimental sepsis study of Mu et al. with lipopolysaccharide-induced acute respiratory distress syndrome in animals, *miR-423-5p* was significantly downregulated and the *FOXA1* gene was upregulated with progression of inflammation and fibrosis [38] (Fig. 6). The positive association of *miR-423-5p* with C-reactive protein suggest a possible link to inflammation, but it remains of uncertain significance [39]. In patients on continuous ambulatory peritoneal dialysis, *miR-423-5p* can serve as a potential biomarker for prediction of cardiac and cerebral events [40]. Thus, the biological impact of *miR-423-5p* is considerable.

### miR-15a-5p

The second most expressed miRNA in our patients was *miR-15a-5p* (Fig. 1), which is recognized as having up to 1415 target genes [27]. In the oATB group, there was a statistically significant change in expression and concentration, with an initial slow downregulation on day 4 and a subsequent upregulation on day 7 (Fig. 2). *miR-15a-5p* is involved in inflammation during sepsis by activating the NF-κB pathway via lipopolysaccharides and targeting negative regulation of the *TNFAIP3* interacting protein 2 gene*.* The result of NF-κB pathway activation is the production of inflammatory cytokines, including TNF-α, IL-1β, and IL-6 [41]. The NF-κB signaling pathway is part of the innate immune defense response via pathogen-triggered activation of Toll-like receptors on the cell membrane. NF-κB activation also requires phosphorylation and degradation of inhibitory κB proteins, which is induced by specific kinases (IKK-α and IKK-β) [42]. *miR-15a-5p* is negatively involved in the NF-ΚB signaling pathway and septic AKI development because of induction of apoptosis in renal cells [43]. However, *miR-15a* can potentially promote NF-ΚB signaling by negatively regulating IKK-α and inhibit genes encoding vascular endothelial growth factor (VEGF)A, VEGFC, and myosin light chain kinase, increasing vascular permeability in sepsis [14] (Fig. 6). In a study involving 166 patients with sepsis and 32 with SIRS, *miR-15a* expression levels were higher with both sepsis and SIRS compared to unaffected participants. Furthermore, *miR-15a* can allow for a distinction between sepsis and SIRS, showing higher expression in SIRS and having a higher AUC for sepsis diagnosis than do C-reactive protein and PCT [44]. Accordingly, with sepsis and the development of septic shock, *miR-15a* expression decreases along with *miR-27a* expression [45]. Both *miR-15a* and *miR-16* are encoded in the same chromosomal region (13q14.3) and experimentally recognized to take part in regulation and decrease of macrophage-mediated phagocytosis in bacterial infection by targeting Toll-like receptor 4–associated pathways, which can aggravate sepsis. However, the expression of *miR-15a* and *miR-16* is different in organs and macrophages and can have both beneficial and detrimental effects [46]. According to our results, circulating *miR-15a-5p* expression did not correspond with changes in primarily investigated inflammatory or renal biochemical variables. However, an important finding of our study is the possibility of using *miR-15a-5p* as a biomarker for AKI in sepsis treated by gentamicin, where in AKI patients was statistically significantly lower (Figs. 4, 5). The pathogenesis of sepsis-induced AKI may involve the *miR-15a-5p*–*X-inactive specific transcript –* Cullin 3 regulatory axis with induction of renal cell apoptosis, according to earlier findings [43].

### miR-155-5p

Circulating *miR-155-5p*, with up to 701 known target genes [27] plays a substantial role in many pathways leading to sepsis and renal injury, with a positive correlation between *miR-155-5p* activation and expression of the pro-inflammatory cytokines IL-6 and IL-8 [47]. An increase in *miR-155-5p* expression also has been observed in experimental I/R injury and in gentamicin nephrotoxicity. However, with a higher dose of gentamicin, *miR-155-5p* levels have been reported to decrease in the urine of experimental animals and increase in renal parenchyma [22]. In our study, we detected no statistically significant association between *miR-155-5p* and any biochemical parameters in the gentamicin group. However, circulating *miR-155-5p* concentrations positively correlated with S_crea_ and NGAL on day 4 in the vancomycin group and with IL-6 and NGAL in all patients. Furthermore, in current study we found positive correlation between *miR-155-5p* and all biochemical markers (PCT, IL-6, NGAL and S_crea_) in all patients together on day 7 (Table 6). Of interest, according to an animal study by Glineur et al., the expression of *miR-155-5p* was significantly dysregulated after administration of puromycin, suggesting that this miRNA could be a candidate biomarker of urinary renal injury [48]. In experimental septic conditions, the inhibition of *miR-155-5p* in animals ameliorates lipopolysaccharide-induced acute lung injury by regulating the *miR-155-5p–*interferon regulatory factor 2 binding protein 2*–*nuclear factor of activated T cells 1 axis [49]. According to study of Kugler et al., the upregulation of *miR-155-5p* in relation to C-reactive protein reflects its established role in inflammation presented on human liver cohort (150 patients) with benign liver diseases. Moreover, *miR-155-5p* pleiotropic functionality was represented by its participation in the active downregulation of ADME (drug absorption, distribution, metabolism and excretion) genes [50].

In addition to nephrological disorders, which can be associated with up- or down-expression of *miR-155-5p*, the plasma concentration of *miR-155-5p* is increased in patients with chronic kidney disease, with significantly higher expression in those with hypertension [51]. *miR-155-5p* is also highly expressed in fibrotic renal tissues, regulates the phosphorylation of *STAT3*, and targets genes encoding suppressor of cytokine signaling 1 and 6, which regulate the profibrotic function of *miR-155-5p* [52].

### miR-192-5p

The fourth miRNA we assessed here, *miR-192-5p*, has up to 219 known target genes [27] and plays a substantial role in biochemical processes associated with I/R injury and vancomycin nephrotoxicity. In experimental I/R renal injury, its expression is significantly upregulated, and *miR-192-5p* has been identified as a potential AKI biomarker [53]. In vancomycin-induced nephrotoxicity, a significant role for *miR-192-5p* upregulation in induction of renal cell apoptosis has been demonstrated [16]. In our study, we found a significant correlation of *miR-192-5p* with PCT, S_crea_, and NGAL on day 7 in the vancomycin group, in accordance with previous findings, and also between *miR-192-5p* and IL-6 on day 4 and PCT and IL-6 on day 7 in the oATB group and with NGAL on day 4 and  PCT in all patients together on day 7 (Table 6). In addition to sepsis and upregulation of pro-inflammatory cytokine (IL-6, IL-1ß, and TNF-α) production, the association of *miR-192-5p* with regulation of the *miR-192-5p*–X-linked inhibitor of apoptosis axis in this condition has been investigated in animals [54] (Fig. 6). Of interest, in an experimental study, Jeon et al. demonstrated a significant upregulation of *miR-192-5p* in gentamicin-induced renal toxicity, coinciding with the appearance of proximal tubular cell necrosis [55]. In an experimental study of cisplatin-induced nephrotoxicity, Kanki et al. found that *miR-192-5p* expression, along with three other miRNAs, significantly correlated with renal injury markers including serum urea, S_crea_, and urinary kidney injury molecule-1 [56]. In 2021, Ren et al. published a comprehensive review of an emerging role of *miR-192-5p* in human diseases including digestive, lung, renal, reproductive, endocrine, and nervous system conditions [57].

#### The current study limitations

The presented study was performed on a relatively small group of critically ill patients with sepsis, which carries a high risk for a type II error (false-negative findings). Furthermore, we did not validate our results in an independent healthy human cohort.

## Conclusions

To the best of our knowledge, this study is the first to assess the time course of expression of selected miRNAs in critically ill patients with sepsis during 7 days of antibiotic treatment. Of the four investigated miRNAs — *miR-15a-5p*, *miR-155-5p*, *miR-192-5p*, and *miR-423-5p —*exhibited similar patterns of individual expression, regardless of antimicrobial therapy and timing. Compared to these two, *miR-423-5p* and *miR-15a-5p* showed significantly higher expression during the 7-day observation. The most expressed in all our patients was *miR-423-5p*, which targets genes associated with septic shock, ischemia, and organ hypoperfusion. *miR-15a-5p* significantly differed between patients with and without AKI in the gentamicin group on day 7 and may have predictive value as a biomarker of AKI in patients with sepsis. *miR-192-5p* and *miR-155-5p* positively correlated with renal injury markers in the vancomycin group. Furthermore, *miR-155-5p* and *miR-423-5p* positively correlated with NGAL on day 4 and with all selected inflammatory and renal injury markers in whole group of patients by day 7. *miR-192-5p* and *miR-423-5p* showed a positive relationship with the inflammation markers PCT and IL-6 in the oATB group, and *miR-192-5p* positively correlated with PCT in the vancomycin group and in all patients on day 7. Therefore, in patients with sepsis treated by potentially nephrotoxic therapy (vancomycin or gentamicin), *miR-423-5p*, *miR-155-5p*, *miR-15a-5p* and *miR-192-5p* may have potential as diagnostic or therapeutic tools in renal injury. More extensive clinical and experimental studies are needed.

## Data Availability

The datasets used and/or analyzed during the current study are available from the corresponding author on reasonable request.

## Abbreviations

AKI: Acute kidney injury
ATB: Antibiotic
miRNA: microRNA

## References

[1] Singer M, Deutschman CS, Seymour CW, Shankar-Hari M, Annane D, Bauer M (2016). The third international consensus definitions for Sepsis and septic shock (Sepsis-3). JAMA..

[2] Bagshaw SM, George C, Bellomo R (2008). ANZICS database management committee. Early acute kidney injury and sepsis: a multicentre evaluation. Crit Care.

[3] Ronco C, Bellomo R, Kellum JA (2019). Acute kidney injury. Lancet..

[4] O'Connor ME, Prowle JR (2015). Fluid Overload. Crit Care Clin.

[5] Kidney Disease: Improving Global Outcomes (KDIGO) Acute Kidney Injury Work Group (2012). KDIGO clinical practice guideline for acute kidney injury. Kidney Inter.

[6] Ma S, Evans RG, Iguchi N, Tare M, Parkington HC, Bellomo R (2019). Sepsis-induced acute kidney injury: a disease of the microcirculation. Microcirculation..

[7] Hoste EA, Bagshaw SM, Bellomo R, Cely CM, Colman R, Cruz DN (2015). Epidemiology of acute kidney injury in critically ill patients: the multinational AKI-EPI study. Intensive Care Med.

[8] Pannu N, Nadim MK (2008). An overview of drug-induced acute kidney injury. Crit Care Med.

[9] Rybak MJ, Lomaestro BM, Rotschafer JC, Moellering RC, Craig WA, Billeter M (2009). Therapeutic monitoring of vancomycin in adults summary of consensus recommendations from the American Society of Health-System Pharmacists, the Infectious Diseases Society of America, and the Society of Infectious Diseases Pharmacists. Pharmacotherapy..

[10] Zamoner W, Prado IRS, Balbi AL, Ponce D (2019). Vancomycin dosing, monitoring and toxicity: critical review of the clinical practice. Clin Exp Pharmacol Physiol.

[11] Hanrahan TP, Kotapati C, Roberts MJ, Rowland J, Lipman J, Roberts JA (2015). Factors associated with vancomycin nephrotoxicity in the critically ill. Anaesth Intensive Care.

[12] Wargo KA, Edwards JD (2014). Aminoglycoside-induced nephrotoxicity. J Pharm Pract.

[13] Caserta S, Kern F, Cohen J, Drage S, Newbury SF, Llewelyn MJ (2016). Circulating plasma microRNAs can differentiate human Sepsis and systemic inflammatory response syndrome (SIRS). Sci Rep.

[14] Giza DE, Fuentes-Mattei E, Bullock MD, Tudor S, Goblirsch MJ, Fabbri M (2016). Cellular and viral microRNAs in sepsis: mechanisms of action and clinical applications. Cell Death Differ.

[15] Benz F, Roy S, Trautwein C, Roderburg C, Luedde T (2016). Circulating MicroRNAs as biomarkers for Sepsis. Int J Mol Sci.

[16] Chen J, Wang J, Li H, Wang S, Xiang X, Zhang D (2016). p53 activates miR-192-5p to mediate vancomycin induced AKI. Sci Rep.

[17] Wang J, Li H, Qiu S, Dong Z, Xiang X, Zhang D (2017). MBD2 upregulates miR-301a-5p to induce kidney cell apoptosis during vancomycin-induced AKI. Cell Death Dis.

[18] Zhou X, Qu Z, Zhu C, Lin Z, Huo Y, Wang X (2016). Identification of urinary microRNA biomarkers for detection of gentamicin-induced acute kidney injury in rats. Regul Toxicol Pharmacol.

[19] Nassirpour R, Mathur S, Gosink MM, Li Y, Shoieb AM, Wood J (2014). Identification of tubular injury microRNA biomarkers in urine: comparison of next-generation sequencing and qPCR-based profiling platforms. BMC Genomics.

[20] Ge QM, Huang CM, Zhu XY, Bian F, Pan SM (2017). Differentially expressed miRNAs in sepsis-induced acute kidney injury target oxidative stress and mitochondrial dysfunction pathways. PLoS One.

[21] Schlosser K, McIntyre LA, White RJ, Stewart DJ (2015). Customized internal reference controls for improved assessment of circulating MicroRNAs in disease. PLoS One.

[22] Saikumar J, Hoffmann D, Kim TM, Gonzalez VR, Zhang Q, Goering PL (2012). Expression, circulation, and excretion profile of microRNA-21, −155, and -18a following acute kidney injury. Toxicol Sci.

[23] Tacke F, Roderburg C, Benz F, Cardenas DV, Luedde M, Hippe HJ (2014). Levels of circulating miR-133a are elevated in sepsis and predict mortality in critically ill patients. Crit Care Med.

[24] Shang W, Wang Z (2017). The update of NGAL in acute kidney injury. Curr Protein Pept Sci.

[25] Ríos-Toro JJ, Márquez-Coello M, García-Álvarez JM, Martín-Aspas A, Rivera-Fernández R, Sáez de Benito A, Girón-González JA (2017). Soluble membrane receptors, interleukin 6, procalcitonin and C reactive protein as prognostic markers in patients with severe sepsis and septic shock. PLoS One.

[26] Brozmanová H, Kacířová I, Uřinovská R, Šištík P, Grundmann M (2017). New liquid chromatography-tandem mass spectrometry method for routine TDM of vancomycin in patients with both normal and impaired renal functions and comparison with results of polarization fluoroimmunoassay in light of varying creatinine concentrations. Clin Chim Acta.

[27] miRbase.org. http://www.mirbase.org. Available online. Accessed on 14 Mar 2021.

[28] R Core Team (2020). R: a language and environment for statistical computing.

[29] Mohammed A, Cui Y, Mas VR, Kamaleswaran R (2019). Differential gene expression analysis reveals novel genes and pathways in pediatric septic shock patients. Sci Rep.

[30] Tirado-Hurtado I, Fajardo W, Pinto JA (2018). DNA damage inducible transcript 4 gene: the switch of the metabolism as potential target in Cancer. Front Oncol.

[31] GENE - NCBI. available online on: www.ncbi.nlm.nih.gov, accessed: in 16 Feb 2021.

[32] Yuan XP, Liu LS, Chen CB, Zhou J, Zheng YT, Wang XP (2017). MicroRNA-423-5p facilitates hypoxia/reoxygenation-induced apoptosis in renal proximal tubular epithelial cells by targeting GSTM1 via endoplasmic reticulum stress. Oncotarget..

[33] Nabiałek E, Wańha W, Kula D, Jadczyk T, Krajewska M, Kowalówka A (2013). Circulating microRNAs (miR-423-5p, miR-208a and miR-1) in acute myocardial infarction and stable coronary heart disease. Minerva Cardioangiol.

[34] Jäntti T, Segersvärd H, Tolppanen H, Tarvasmäki T, Lassus J, Devaux Y (2019). Circulating levels of microRNA 423-5p are associated with 90 day mortality in cardiogenic shock. ESC Heart Fail.

[35] Bruno N, ter Maaten JM, Ovchinnikova ES, Vegter EL, Valente MA, van der Meer P (2016). MicroRNAs relate to early worsening of renal function in patients with acute heart failure. Int J Cardiol.

[36] Mårtensson J, Bell M, Oldner A, Xu S, Venge P, Martling CR (2010). Neutrophil gelatinase-associated lipocalin in adult septic patients with and without acute kidney injury. Intensive Care Med.

[37] Barbagallo C, Passanisi R, Mirabella F, Cirnigliaro M, Costanzo A, Lauretta G (2019). Upregulated microRNAs in membranous glomerulonephropathy are associated with significant downregulation of IL6 and MYC mRNAs. J Cell Physiol.

[38] Mu X, Wang H, Li H (2021). Silencing of long noncoding RNA H19 alleviates pulmonary injury, inflammation, and fibrosis of acute respiratory distress syndrome through regulating the microRNA-423-5p/FOXA1 axis. Exp Lung Res.

[39] Bretthauer J, Anker SD, Pinet F, Thum T (2013). Circulating miR-133a and miR-423-5p fail as biomarkers for left ventricular remodeling after myocardial infarction. Int J Cardiol.

[40] Wang Y, Liu C, Wei W, Chen W (2020). Predictive value of circulating coagulation related microRNAs expressions for major adverse cardiac and cerebral event risk in patients undergoing continuous ambulatory peritoneal dialysis: a cohort study. J Nephrol.

[41] Lou Y, Huang Z (2020). microRNA-15a-5p participates in sepsis by regulating the inflammatory response of macrophages and targeting TNIP2. Exp Ther Med.

[42] Kawai T, Akira S (2007). Signaling to NF-kappaB by toll-like receptors. Trends Mol Med.

[43] Xu G, Mo L, Wu C, Shen X, Dong H, Yu L (2019). The *miR-15a-5p-XIST-CUL3*regulatory axis is important for sepsis-induced acute kidney injury. Ren Fail.

[44] Wang H, Zhang P, Chen W, Feng D, Jia Y, Xie LX (2012). Evidence for serum miR-15a and miR-16 levels as biomarkers that distinguish sepsis from systemic inflammatory response syndrome in human subjects. Clin Chem Lab Med.

[45] Goodwin AJ, Guo C, Cook JA, Wolf B, Halushka PV, Fan H (2015). Plasma levels of microRNA are altered with the development of shock in human sepsis: an observational study. Crit Care.

[46] Moon HG, Yang J, Zheng Y, Jin Y (2014). miR-15a/16 regulates macrophage phagocytosis after bacterial infection. J Immunol.

[47] Pfeiffer D, Roßmanith E, Lang I, Falkenhagen D (2017). miR-146a, miR-146b, and miR-155 increase expression of IL-6 and IL-8 and support HSP10 in an in vitro sepsis model. PLoS One.

[48] Glineur SF, Hanon E, Dremier S, Snelling S, Berteau C, De Ron P (2018). Assessment of a urinary kidney MicroRNA panel as potential nephron segment-specific biomarkers of subacute renal toxicity in preclinical rat models. Toxicol Sci.

[49] Li HF, Wu YL, Tseng TL, Chao SW, Lin H, Chen HH (2020). Inhibition of miR-155 potentially protects against lipopolysaccharide-induced acute lung injury through the IRF2BP2-NFAT1 pathway. Am J Physiol Cell Physiol.

[50] Kugler N, Klein K, Zanger UM (2020). MiR-155 and other microRNAs downregulate drug metabolizing cytochromes P450 in inflammation. Biochem Pharmacol.

[51] Klimczak D, Kuch M, Pilecki T, Żochowska D, Wirkowska A, Pączek L (2017). Plasma microRNA-155-5p is increased among patients with chronic kidney disease and nocturnal hypertension. J Am Soc Hypertens.

[52] Zhang W, Li X, Tang Y, Chen C, Jing R, Liu T (2020). miR-155-5p implicates in the pathogenesis of renal fibrosis via targeting SOCS1 and SOCS6. Oxidative Med Cell Longev.

[53] Zou YF, Wen D, Zhao Q, Shen PY, Shi H, Zhao Q (2017). Urinary MicroRNA-30c-5p and MicroRNA-192-5p as potential biomarkers of ischemia-reperfusion-induced kidney injury. Exp Biol Med (Maywood).

[54] Sun F, Yuan W, Wu H, Chen G, Sun Y, Yuan L (2020). LncRNA KCNQ1OT1 attenuates sepsis-induced myocardial injury via regulating miR-192-5p/XIAP axis. Exp Biol Med (Maywood)..

[55] Jeon BS, Lee SH, Hwang SR, Yi H, Bang JH, Tham NTT (2020). Identification of urinary microRNA biomarkers for *in vivo* gentamicin-induced nephrotoxicity models. J Vet Sci.

[56] Kanki M, Moriguchi A, Sasaki D, Mitori H, Yamada A, Unami A (2014). Identification of urinary miRNA biomarkers for detecting cisplatin-induced proximal tubular injury in rats. Toxicology..

[57] Ren FJ, Yao Y, Cai XY, Fang GY (2021). Emerging role of MiR-192-5p in human diseases. Front Pharmacol.

